# Antibodies in action: the role of humoral immunity in the fight against atherosclerosis

**DOI:** 10.1186/s12979-022-00316-6

**Published:** 2022-12-02

**Authors:** Joshua A. Taylor, Mark A. Hutchinson, Patricia J. Gearhart, Robert W. Maul

**Affiliations:** 1 Laboratory of Molecular Biology and Immunology, National Institute on Aging, NIH, Baltimore, MD USA; 2 Graduate Program in Immunology, Johns Hopkins University School of Medicine, Baltimore, MD USA

**Keywords:** AID, Atherosclerosis, B cells, Antibodies

## Abstract

The sequestering of oxidation-modified low-density lipoprotein by macrophages results in the accumulation of fatty deposits within the walls of arteries. Necrosis of these cells causes a release of intercellular epitopes and the activation of the adaptive immune system, which we predict leads to robust autoantibody production. T cells produce cytokines that act in the plaque environment and further stimulate B cell antibody production. B cells in atherosclerosis meanwhile have a mixed role based on subclass. The current model is that B-1 cells produce protective IgM antibodies in response to oxidation-specific epitopes that work to control plaque formation, while follicular B-2 cells produce class-switched antibodies (IgG, IgA, and IgE) which exacerbate the disease. Over the course of this review, we discuss further the validation of these protective antibodies while evaluating the current dogma regarding class-switched antibodies in atherosclerosis. There are several contradictory findings regarding the involvement of class-switched antibodies in the disease. We hypothesize that this is due to antigen-specificity, and not simply isotype, being important, and that a closer evaluation of these antibodies’ targets should be conducted. We propose that specific antibodies may have therapeutical potential in preventing and controlling plaque development within a clinical setting.

## Background

Heart disease is the leading cause of death in both the United States and internationally, with cases escalating in recent decades. A vast majority of these deaths are associated with atherosclerosis, the manifestation of fatty plaques within arterial walls, and is estimated to cost Americans over 300 billion annually in healthcare fees [1]. The accumulation of these fatty plaques affect large and medium-sized arteries alike, and leads to many life-threatening clinical complications, including stroke, cardiac infarction, and ischemic damage to the kidneys [2].

**Fig. 1:**
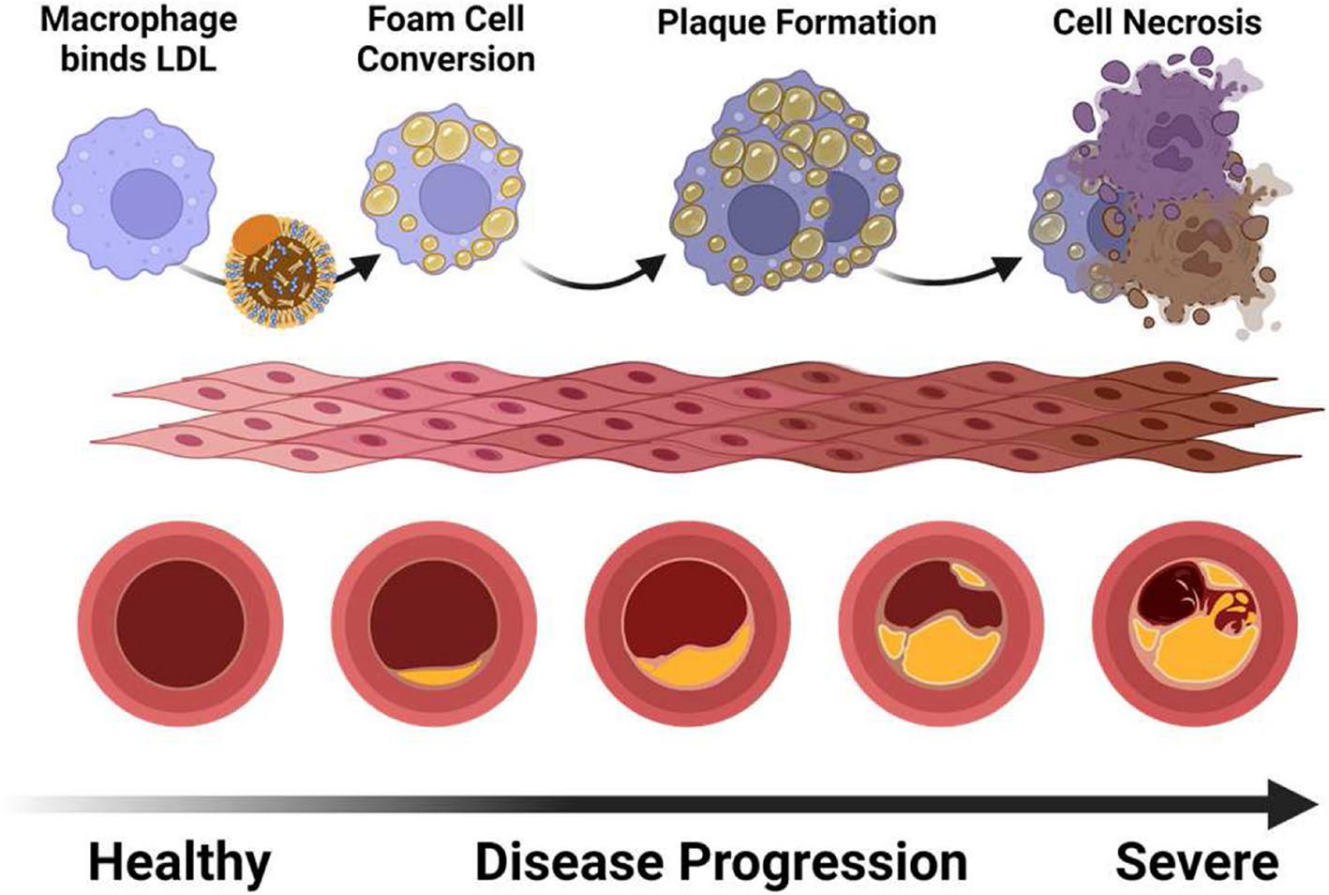
Atherosclerosis begins with the recognition of low-density lipoprotein by scavenger receptors on monocytes and macrophages. These cells infiltrate into the intimal layer of the artery where they uptake these lipid-rich molecules. Inability to process the heavy lipid burden overwhelms the cells and causes conversion to a foam cell. As foam cells accumulate, plaques develop, leading to further occlusion of arteries. In late-stage plaques, the hypoxic environment promotes necrosis of macrophages, smooth muscle cells, and the endothelial lining

Plaque accumulation is associated with an elevation in total circulating cholesterol and an imbalance between low-density lipoprotein (LDL) and high-density lipoprotein (HDL) levels [3–6]. Accumulation of LDL within the intimal layer of the major arteries allows these molecules to be targeted by reactive oxygen species, generating oxidation-specific epitopes (OSEs) that are recognized by scavenger receptors present on macrophages [7–9]. This binding triggers endocytosis of the lipid-rich substrates by the macrophage, with incessant rounds of LDL engulfment overwhelming the digestive machinery of the cell and converting the macrophage into a foam cell [10, 11]. Continuous infiltration of macrophage-polarized monocytes into the plaque, and their conversion into foam cells, eventually fosters an environment that subjugates and impairs the proper clearance of apoptotic cells within the environment through means such as effectorcytosis. Elevated necroptosis, a non-apoptotic form of cell death, of the macrophages and smooth muscle cells present within these lesions further compounds this issue [12]. This results in necrotic core formation, destabilization of the plaque, and increases release of inflammasome-mediated cytokines that promote sterile inflammation [13, 14]. This pathway causes modified degradation of intracellular components [15], generating end products which upon core rupture spill into the blood alongside cellular debris recognized by the innate immune system as danger-associated molecular patterns (DAMPs) [16]. These two components together have the potential of generating adaptive responses against autoantigens and worsening the arterial inflammation (Fig. 1).

Over the course of this review, we will discuss how lymphocytes of the adaptive immune response are involved throughout this process and focus on the role that the antibody responses play in atherosclerosis disease progression and prevention.

### T lymphocytes

**Fig. 2:**
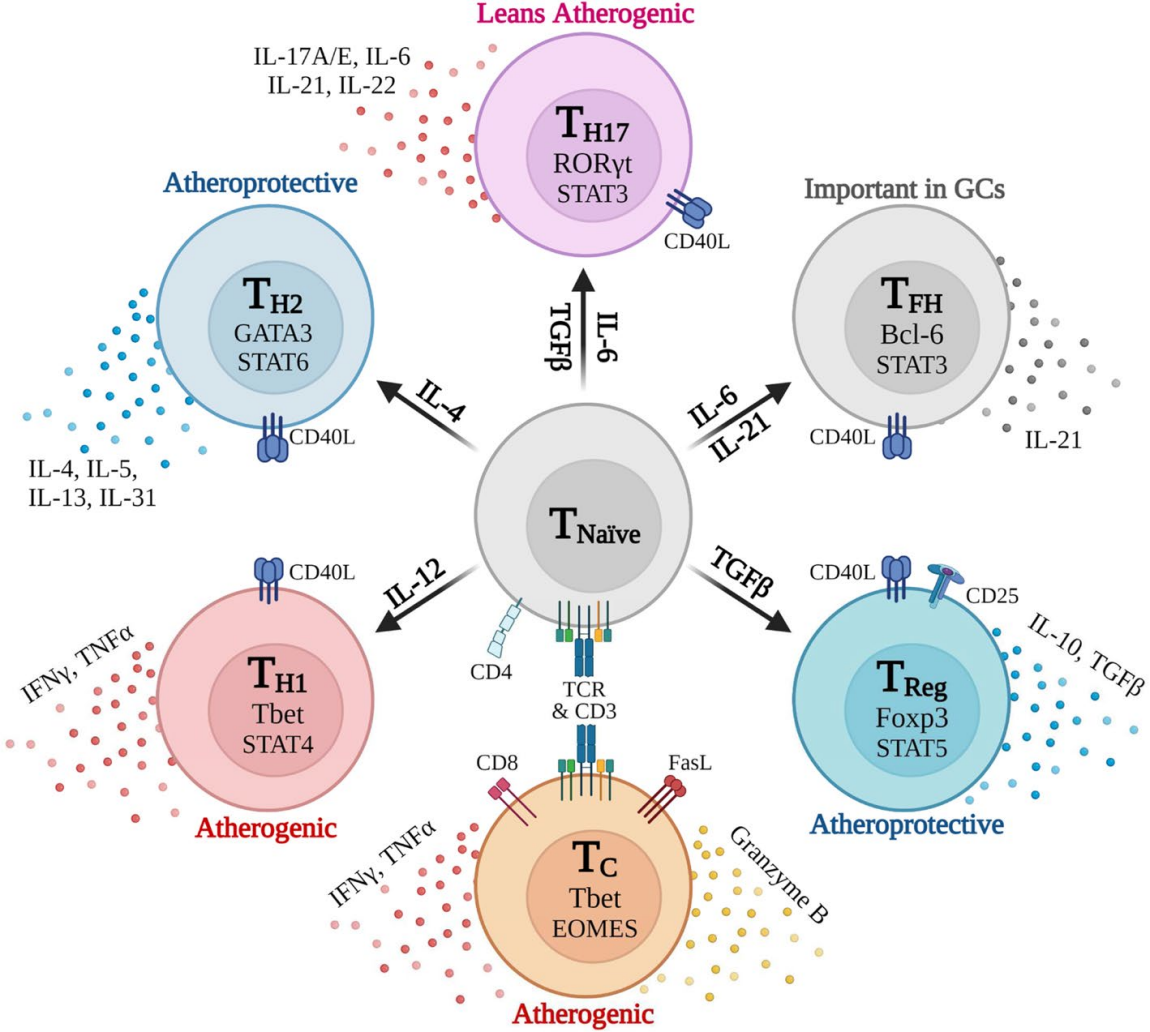
T cells contribute to the development and progression of atherosclerosis in a subset-dependent manner. CD4^+^ T cells begin as inexperienced naïve cells, upon activation through their TCR via interaction with MHC, polarize to a specific activated population due to signals induced by cytokines present within their surrounding microenvironment. Following activation, these cells secrete additional cytokines and express elevated levels of CD40L on their surface providing T cell help to B cells and other immune cells. CD8^+^ T cells function through the secretion of cytokines and conduct immunosurveillance, where they can induce cell death in target cells in a TCR-dependent manner using Granzyme B and Fas-FasL signaling

In addition to macrophage, T cells are also found within arterial plaques. T cells are a major subpopulation of the adaptive immune system and are considered key drivers in the response to foreign and altered-self epitopes. These cells undergo development in the thymus generating a plethora of T cells all containing a unique T cell receptor (TCR) which regulate the antigen specificity of the cell. T cells respond to peptides presented by cells in the context of Major Histocompatibility Complexes (MHC) through engagement with the T cell’s TCR [17–19]. The TCR-MHC interaction initiates cellular responses, the production of cytokines, and interaction with other immune cells, including B cells, in order to dictate the nature of the generated response (Fig. 2).

The contribution these cells play in the development of atherosclerosis has been a major focus of research for the past several decades. T cells are seen in the early formation of the plaque environments and have a strong presence throughout many stages of lesion development in humans and mice [20–23]. Likewise, knocking out these cells by deletion of the Rag Recombinase, which ablates TCR development, results in decreased plaque burden in atherosclerotic *ApoE*-deficient mice [24, 25]. It should be noted that Rag-deficiency also inhibits B cells development in these animals. T cell expression of CD40L (CD154) provides signals needed for the proper activation of many cytotoxic T cells and B cells through engagement with CD40. This CD40-CD40L interaction is also involved in the effector function of other non-lymphocyte cells, such as macrophages, which has been reviewed elsewhere [26–28].

#### CD8^+^ cytotoxic T cells

T lymphocytes can be divided into two major functional subgroups: CD8^+^ cytotoxic T cells, which bind MHC Class I expressed ubiquitously on cells, and CD4^+^ helper T cells, which bind MHC Class II expressed almost exclusively on antigen-presenting cells (APCs). The role of CD8^+^ T cells in atherosclerosis is poorly understood. CD8^+^ T cells have been shown to be present within early plaques, and antibody depletion of CD8^+^ T cells exhibits decreased plaque burden in atherosclerotic *ApoE*^*−/−*^ and *LDLR*^*−/−*^ mice. However, similar levels of CD8^+^ T cell infiltrate into the aorta of *ApoE*^*−/−*^ mice regardless of a high-fat diet, suggesting a disconnect between recruitment and disease severity [29–31]. In humans, higher CD8^+^ cells with low CD127 and higher PD-1 expression has been observed in the plaques of patients with cardiovascular disease who have experienced a recent stroke, suggesting that CD8^+^ effector cells might be involved in the comorbidities of the disease and become exhausted following the cardiovascular event [32].

#### CD4^+^ effector T cells

By comparison, CD4^+^ T cells in atherosclerosis are better characterized, but display a more complex relationship with the disease due to the different possible subtypes of the CD4^+^ T cell (Fig. 2). CD4^+^ T cells are divided into several major subclasses based on master transcription factor expression and the cytokines they secrete. Type 1 T Helper cells (T_H_1) express Tbet and produce high levels of IFNγ upon activation. IFNγ activates macrophages, increasing their inflammatory potential, and during atherosclerosis has a potent proatherogenic effect on plaque formation [33–36] with deletion of Tbet and IFNγ alleviating this plaque formation in *ApoE*^*−/−*^ mice [37, 38]. By comparison, T_H_2 cells, characterized by their expression of GATA3 and production of IL-4, IL-5, IL-13, and IL-33 are considered to be more anti-atherogenic; although their actual contribution is controversial. IL-4 produced may promote atherosclerosis [39–43], while IL-5 and IL-33 provides protection by promoting atheroprotective antibody production against malondialdehyde-modified LDL [44, 45]. RORγT-expressing T_H_17 cell involvement is likewise controversial due to the inconsistencies regarding the effect of IL-17A. IL-17A correlates with worsening disease prognosis in humans and mice, and blocking IL-17A with antibodies decreases plaque burden in *ApoE*^*−/−*^ mice. However, IL-17A deficiency also causes increased plaque instability, suggesting a differential effect in early compared to late-stage disease [46–49]. CD25^+^, Foxp3-expressing T Regulatory (Treg) cells, which secrete TGFβ and IL-10, are widely accepted as atheroprotective by inhibiting atherogenic T Cells and macrophages [50, 51]. It has been proposed that Tregs are important for controlling atherosclerosis in early- and mid-stage plaques, and failure to maintain a high Treg-to-effector ratio results in advanced plaque formation of clinical concern [52–59]. This is further supported by the discovery that responding Tregs from the early stages of plaque formation can undergo transcriptional changes and emerge as T_H_1 and T_H_17 effectors cells in late-stage lesions [60].

### B lymphocytes

Compared to T cells, the history of research on B cell involvement in atherosclerosis has had a short life, picking up only in the past few decades. Unlike T lymphocytes, B cells produce antibodies that disseminate throughout the body independent of the cell itself. As a result, B cells are not as dependent on localization to the direct site of inflammation with B cells making up only a minute proportion of the cells infiltrating the early plaque environment [31, 61]. Conversely, late-stage plaques show an oligoclonal expansion of B cells within the affected atherosclerotic tissue with reports of artery tertiary lymphoid structures (ATLOs) developing and producing antibodies within the adventitial layer of a diseased aorta [62–68]. Other B cells likewise can be found within the perivascular adipose tissue (PVAT) surrounding the aorta and are present in greater abundance than on the aorta itself [61, 64, 69]. These cells are elevated in areas surrounding plaques, independent of plaque stability, and produce abundant cytokines and antibodies [61, 69]. Compared to B cells of the adventitia, PVAT B cells may be involved in plaque modulation earlier as B cells do not have a high rate of trafficking to the adventitia following adoptive transfer [63]; however, the nature of this may vary due to differential localization of adoptively transferred B cells around the aorta based on subtype [61].

**Fig. 3:**
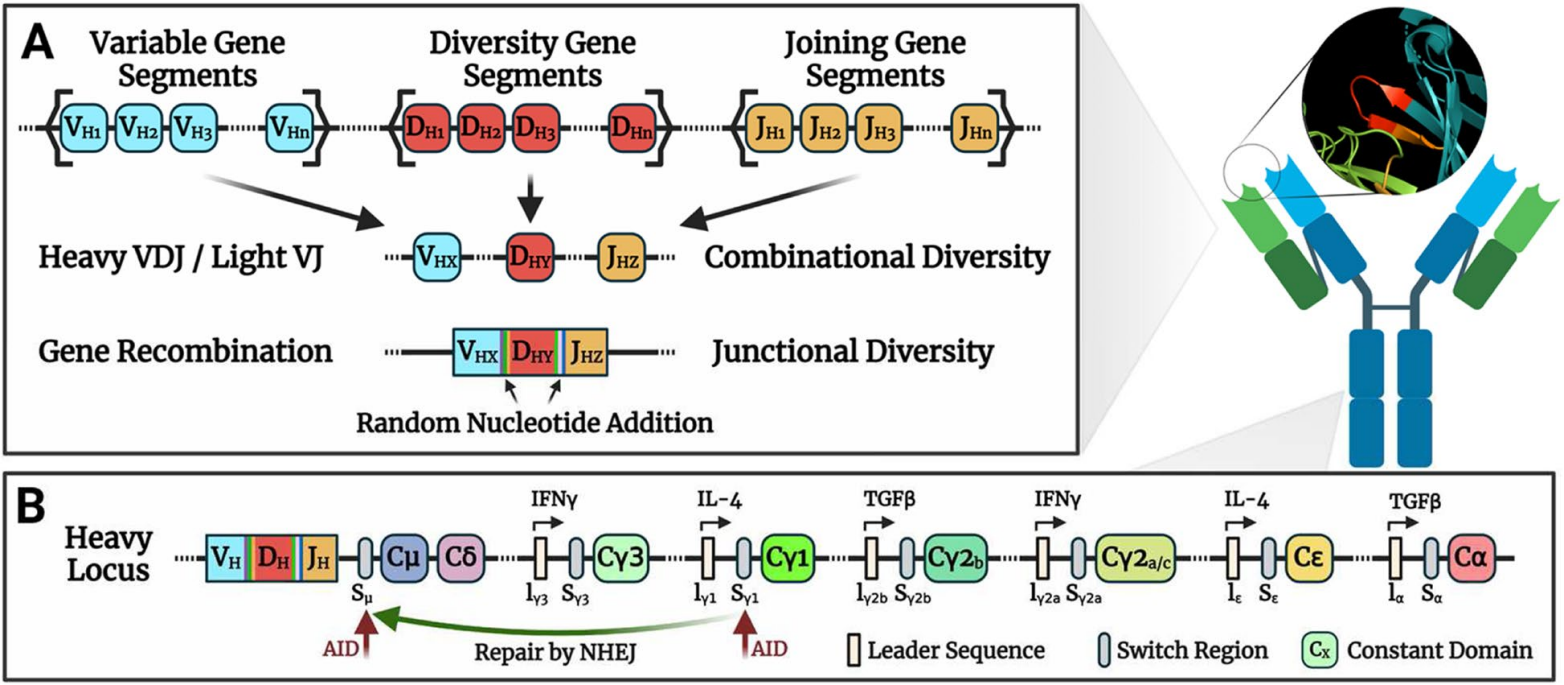
Antibody diversity comes from combination of variable and constant domain gene elements. **A** The B Cell locus is composed of multiple individual Variable, Diversity, and Joining gene segments which are randomly assembled into a functional gene exon through V(D)J recombination. The recombination involves the addition of random nucleotides, increasing the junctional diversity. The sites of the rearrangement form the third complementary determining region of the antibody, which dictates the antigen binding capability of the antibody (as shown in the callout image). **B** Transcription from intron promoters preceding switch regions allows AID to act and induce double stranded breaks. Repair by Nonhomologous end joining (NHEJ) between two broken switch regions results in class switching

With regard to development, B cells start out as hematopoietic stem cells in the bone marrow where they progress through a series of checkpoints dependent on expression of transcription factors E2A, PU.1, and Pax5. Each B cell expresses a unique B cell receptor (BCR) which is derived from two independent peptides, the immunoglobulin heavy chain (IgH) and Ig light chain, with the Ig light chain being expressed from either the Ig kappa (Igκ) or Ig lambda (Igλ) locus. To form a unique BCR, a B cell will recombine each loci through the process of V(D)J recombination (Fig. 3A) [70, 71].

Briefly, V(D)J recombination is the process of bringing a single variable (V), diversity (D), and joining (J) gene segment for IgH, or V and J for Igκ and Igλ, together through genomic recombination. In mice, each loci contains multiple gene segments (3 V and J for Igλ, ~ 80 V and 4 J for Igκ, and ~ 100 V, 13 D, and 4 J for IgH), millions of unique BCR combination can be generated. Through alternative splicing mechanisms, BCRs can be secreted as antibodies, and undergo additional diversity in their V and constant (C) regions after encountering their cognate antigen. Multiple different C regions can be observed across cells sharing the same clonality due to the ability of B cells to undergo the process of class-switching, giving rise to five immunoglobulin classes (IgM, IgD, IgG, IgE, and IgA) with several additional subclasses existing (Fig. 3B). This process is initiated by the activation-induced deaminase (AID) enzyme, which also generates mutations in variable genes to make high affinity antibodies. Following development and further maturation of these cells, self-reactive clones are deleted through the process of negative selection within the bone marrow and the periphery, resulting in immunological tolerance. Failure of this process may occur due to a breakdown of anatomical barriers and the exposure of epitopes that cells would otherwise be ignorant for, or because of improper suppression of clones that become self-reactive as a byproduct of AID-mediated genetic drift. For this reason, the emergence of autoreactive antibodies within atherosclerosis highlights the potential importance of studying these cells and understanding their contribution to the disease.

#### B-1 cells

B cells throughout the body can be divided into two distinct linages, B-1 and B-2 (Table 1), with both populations suspected to provide distinct roles in the progression of atherosclerosis. B-1 cells initially emerge during the period of fetal and neonatal development [72–75] and are the major resident B cell populations of the body’s cavities, such as the peritoneal and plural cavity, where they undergo homeostatic proliferation. B-1 responses are often geared towards evolutionarily conserved epitopes, such as bacterial components and apoptotic debris, functioning independent of T cell signaling [76–82]. The antibodies produced by these cells are primarily IgM, have little junctional nucleotide additions, and have a strong bias for certain V gene segments that compose their BCRs [83, 84]. B-1 cells can be further classified into two additional subpopulations, B-1a and B-1b, which differ based on the expression of CD5. The functional difference between these two cell populations is not fully understood; however, B-1a cells are thought to be the main producers of baseline IgM levels while B-1b cells generate the bulk of neutralizing IgM in response to infection and show class switching to IgA [81, 82, 85–87]. Regardless, both B-1a and B-1b cells are largely considered beneficial in atherosclerosis through their secretion of natural, relatively unmutated IgM directed against OSEs, which aid in the attenuation of plaque development in atherosclerotic *ApoE*^*−/−*^ mice by blocking foam cell development [61, 88–90]. Elevation of these cells, either by knockout of Siglec-G, which negatively regulates B-1 and other myeloid populations and fosters tolerization in these cells [91, 92], or by modulation of TIM-1^+^ B cells using an anti-TIM-1 antibody [93] has moreover been shown to provide increased atheroprotection. It has also been argued that these cells are critical for preventing inflammation-promoting vascular neutrophilia [94, 95]. Recently it has been reported that the human-equivalent of B-1 cells (CD20^+^ CD27^+^ CD38^+^ CD43^+^) decline with age and are stunted in their ability to secrete antibodies, providing a potential mechanism for the acceleration of plaques in aged individuals [96]. Likewise, not all B-1 cells are believed to be protective. A subpopulation of B-1a cells, termed Innate Response Activator (IRA) B cells, have been found that aggravate atherosclerosis in mice through the production of IFNγ and GM-CSF. These cytokines act on cells within the plaque and cause an increase in monocyte recruitment and infiltration, drive the polarization of macrophages towards a M1 phenotype, and promote further CD4^+^ T cell development into inflammatory T_H_1 cells [97–101].

**Table 1:** B cell subtypes and characteristics

	B-1 Cells	B-2 Cells	B-2 Cells
Subset	B-1a, B-1b	Marginal zone (MZ)	Follicular (FO)
Location	Peritoneal cavity, spleen	Spleen, lymph node	Spleen, lymph node
Major Ig	IgM, IgA	IgM	IgM, IgG, IgA, IgE
V Genes	Limited V-gene repertoire	Limited V-gene repertoire	Diverse V-gene repertoire
V Gene Mutations	Non-mutated V-genes	Low mutated V-genes	High mutated V-genes (Increased Affinity)

#### B-2 cells

While B-1 cells have been a major focus of atherosclerosis research, B-2 lymphocytes make up most of the B cells present within the body. B-2 cells are found preferentially in the spleen and lymph nodes and possess more diverse BCRs compared to their B-1 counterparts. The B-2 compartment is composed of Marginal Zone (MZ) and Follicular (FO) B cells (Table 1), which differ in their naïve form based of their preferential expression of Cμ or Cδ genes, CD21 and CD23 levels, and roles in response to infections. MZ B cells are IgM^hi^ IgD^lo^ CD21^hi^ CD23^lo^ B lymphocytes which have strong similarities to B-1 cells in terms of function and repertoire diversity [78]. By comparison, FO B cells are IgM^lo^ IgD^hi^ CD21^lo^ CD23^hi^ B cells, and show a greater assortment of BCR sequences between clones, allowing the FO cells to bind to a larger array of potential epitopes. Upon activation, FO B cells are dependent on engaging CD40L on CD4^+^ T cells through CD40 receptor on their cell surface in order to properly activate and produce high titers of antigen-specific antibodies. Upon T cell engagement, FO B cells enter specialized germinal center structures within the spleen and lymph nodes to undergo further diversification. Loss of the CD40/CD40L signaling axis impairs the ability of these cells to generate germinal centers, inhibiting the process of affinity maturation [102]. Furthermore, while evidence proposes that both MZ and FO cells can undergo germinal center formations [103], FO B cells more readily form these complexes upon activation, favoring the production of higher affinity antibodies.

The impact of B-2 cells in atherosclerotic plaque formation is heavily debated, and despite efforts to understand their involvement, the mechanism of their contribution is still unknown. During atherosclerosis, B-2 cells appear to isolate to novel structures within the adventitia of the vessel termed artery tertiary lymphoid organs (ATLOs) [104]. Within the ATLOs, B-2 cells engage T cells and dendritic cells and initiate an adaptive immune response. Reduction of the B cell compartment by splenectomy, reducing B-2 total cell populations, results in increased plaque formation in *ApoE*^*−/−*^ mice, indicating that these B cells play a role in the disease [105]. Similar findings were observed in *LDLR*^*−/−*^ μMT mice, which lack all B cells [106]. Reintroduction of the B-2 compartment into *ApoE*^*−/−*^ *μMT* mice however gives mixed results as two studies claim atherosclerosis is increased [107, 108], while another states the opposite in a Id3-deficent system [109]. B-2 MZ B cells themselves are thought to provide protection against disease through similar mechanisms as B-1 cells and a reported ability to regulate T Follicular Helper (T_FH_) cells through PDL1 signaling [110]. Meanwhile, specific antibody depletion of B-2 cells has also been shown to provide protection [108, 111]. Similar results have been observed by knocking out MHC Class II or CD40, which are required for FO B cell activation [112]. While there are non-B cell effects of CD40 disruption on atherosclerosis [113], B cell specific effects support the detrimental role of B-2 activation in plaque formation [114]. This suggests that B-2 FO cells could have a pro-atherogenic role, which is the current understanding of the field. These studies however utilize broad immunomodulatory methods with some inconsistencies existing within the results, indicating that a more intricate evaluation of B-2 cells and their antibodies could aid our understanding of these cells within the disease.

### Antibodies in action: IgM

Upon successful recombination of a BCR encoding gene, IgM is the first antibody class able to be produced by the B cell prior to activation. IgM is naturally present within the serum where it exists as a pentamer linked together by the Immunological J chain (Ig J) peptide, which regulates the oligomerization of these complexes [115]. This oligomerization is a critical component of IgM’s ability to bind its cognate antigen due to the binding affinity of a single IgM monomer being a considerably weak interaction. The pentameric formation of IgM increases the valency of the complex from 2 potential binding sites to 10, dramatically increasing the avidity of the molecule to multivalent antigens.

Natural IgM is secreted mostly by B-1 and MZ B Cells, which produce these molecules against evolutionary conserved motifs present on pathogen-associated molecular patterns (PAMPs), such as the cell wall components of bacterium. These natural antibodies are produced without the need for T-cell help and can be considered ‘germline’ due to their low mutation levels and ability to be generated regardless of infection [116]. IgM bound to PAMPs is recognized by C1q, which activates the classical pathway of the complement cascade, resulting in the formation of pore complexes on the plasma membrane of pathogens and cell lysis. IgM-bound C1q can also be recognized by an array of C1q receptors, including C1qRp which mediates C1q-mediated phagocytosis by neutrophils and monocytes [117, 118]. IgM may also directly aid in the recognition and clearance of PAMPs and DAMPs through opsonization and uptake of IgM-antigen complexes by Fcα/μR on phagocytic B cells and monocytes [119], which also negatively regulates humoral responses for T-independent antigens [120]. IgM is likewise recognized by FcμR, expressed on B cells in mice and B, T, and NK lymphocytes in humans, which mediates developing B cell activation and subtype distribution [121, 122], and is responsible for regulating IgM titers [123]. Interestingly, soluble IgM has been reported to be involved in the suppression of autoantibody production [124], with FcμR possibly mediating this function [125], thus making this IgM-FcμR interaction a potential interest for atherosclerosis research.

#### Atheroprotective nature of IgM

The IgM routinely observed in atherosclerosis is widely accepted to have an atheroprotective role in the prevention and attenuation of the disease in humans [126, 127] and directly in mice [128]. This protection is dependent on IgM secretion as secretion-deficient IgM (*sIgM*^*−/−*^) mice, which lack serum IgM due to the deletion of an exon in the Cμ gene locus, showed exacerbated plaque lesions [129]. While other IgM molecules may also provide protection in the diseases, IgM generated in response to the modification of biomolecules by reactive oxygen species, termed oxidation-specific epitopes (OSE), has been the most studied and these molecules are commonly found within the serum of mice and humans [130]. OSE specific IgM show a strong correlation with the control of atherosclerotic plaque formation across several human studies [130, 131].

In atherosclerosis, antibodies against oxidized forms of low-density lipoprotein (OxLDL) have been of great interest. The term OxLDL is used to encompass several different modifications of the LDL molecule with malondialdehyde (MDA), malondialdehyde-acetaldehyde (MAA), and 4-hydroxynonenal (4HNE) being the most well studied. These modifications are observed on ApoB100 and other proteins of the LDL molecule. Oxidation of phospholipids has also been observed on the LDL molecule, specifically lipid adducts (POVPC, PGPC, PEIPC, etc.) which mimic the structure of phosphorylcholine (PC) [132, 133] and play an important role in early atherogenesis in *LDLR*^*−/−*^ mice by mediating monocyte binding [134]. Immunization with oxLDL, namely MDA-OxLDL, has been shown to provide protection in atherosclerotic mice with high titers of IgM against these adducts being observed [135–137]. Responses to these LDL OSEs have since been shown to be important in preventing toxicities caused by release of additional reactive oxygen species [138–143].

In 1996, the Witztum group generated an IgM-producing hybridoma, E06, which showed ability to bind oxidized phospholipid and plaques within atherosclerotic rabbits [144]. Strikingly, this antibody was found to have an identical peptide sequence to the phosphorylcholine (PC)-binding T15 clone discovered to emerge during fetal development and provide protection from *Streptococcus* infections [145, 146]. This prompted the discovery that antibodies with the E06/T15 heavy and light variable regions, or idiotype, could neutralize CuOxLDL, and upon passive transfer into mice, reduce plaque formation [147, 148]. Similar results were observed following immunization with PC and *S. pneumoniae* which generated high titers of these antibodies [149, 150]. Additionally, several studies have found a correlation between anti-PC antibodies and decreased disease in patients [151].

IgM antibodies are mostly thought to function through three mechanisms; 1) receptor-mediated endocytosis, 2) complement-mediated opsonization, and 3) antigen neutralization, each of which may contribute to the protective effect of IgM within atherosclerosis. Additional work with the E06/T15 antibodies showed that these antibodies readily bind POVPC and PEIPC lipids present on apoptotic cells [147, 152], suggesting these antibodies may aid in clearance of apoptotic cells and debris. T15 IgM promotes dendritic cell phagocytosis and clearance of apoptotic cells within plaques as measured by TUNEL staining [90, 153]. Knockout of MERTK, a receptor tyrosine kinase responsible for recognition of apoptotic cells, in *ApoE*^*−/−*^ mice results in a dramatic increase in necrosis and TUNEL staining; however, plaques volumes are not significantly increased [154], suggesting that IgM:receptor-mediated endocytosis may play a secondary role in terms of IgM in atherosclerosis. Likewise, loss of IgM-mediated opsonization may only play a minor role in the disease as knockout of C1q, which impairs the clearance of apoptotic cells and debris, shows a less pronounced plaque increase compared to *sIgM*^*−/−*^ mice [129]. Based on these findings, we suspect that antigen neutralization may be the major driver of IgM-mediated protection in atherosclerosis as antibodies against OxLDL have been shown to provide significant mediation of atherosclerotic risk by inhibiting macrophage phagocytosis and foam cell formation [11, 130, 155–158]. This protection is likewise observed to be independent of antibody class as IgG-derived Fabs and single chain variable fragments, which lack the constant domains necessary for Fc receptor binding and thus can only function through neutralization, show a robust ability to bind apoptotic debris and prevent macrophage phagocytosis [142, 157]. Recently, a fourth mechanism of protection has been proposed due to the ability of OSE-specific IgM to block the interaction of Factor Xa with microvesicles and prevent coagulation and thrombosis in C57BL/6 J animals [159]. The effort to understand the impact this novel mechanism is still in an early phase, yet this work provides exciting implications for the role of IgM in the disease; particularly, the control of plaques within late-stage lesions and the prevention of necrotic core rupture.

In a recent study done by our group, we observed that *ApoE*^*−/−*^ mice deficient in AID had extensively reduced plaque burden (80%) [160]. The loss of AID results in the inability of B cells to undergo class switch recombination and somatic hypermutation. Consequentially, only low-affinity IgM antibodies can be detected in the serum of these animals and BCR expression is restricted to the Cμ and Cδ genes. This does not change in the distribution of B cell subsets (B-1a, B-1b, MZ, and FO) compared to *ApoE*^*−/−*^ mice. Furthermore, there was no difference in splenic germinal center B cell numbers, suggesting that hyperlipidemia alone is sufficient to drive the formation of these reactions. Interestingly, we observed a dramatic decrease in aortic B cells and a massive increase in MDA-OxLDL IgM in the *ApoE*^*−/−*^*Aid*^*−/−*^ mice compared to *ApoE*^*−/−*^ on a high fat diet. This suggests that AID is somehow impeding proper generation of atheroprotective antibodies. It should be noted that because of the normal mechanisms of V(D)J recombination and negative selection, the *ApoE*^*−/−*^ *Aid*^*−/−*^ IgM will have a more diverse repertoire than B-1-derived IgM, and MZ-derived IgM (Table 1) which could be why these mice can produce higher amounts of OxLDL specific IgM than an *ApoE*^*−/−*^ mouse.

### Mechanism of antibody affinity maturation and class switching

Upon activation of the B cell, antibodies are able to undergo further maturation and the process of class switching. Activated B cells form germinal center structures where they undergo continuous cycling between the light and dark zone based on CXCR4 expression. Antigen engagement with the BCR receptor triggers the expression of CXCR4 on the B cell, which migrates into the dark zone against a CXCL12 chemokine gradient generated by follicular dendric cells [161]. Within the dark zone, B cells undergo several rounds of cell division and express the *Aicda* gene, giving rise to Activation-Induced Deaminase (AID), which introduces mutations into the DNA of the antibody gene and results in changes to the BCR heavy and light chain peptide. These altered B cell clones migrate out of the dark zone into the light zone where they compete for antigen and CD40 ligand signaling from T Follicular Helper (T_FH_) cells. The binding of these elements are required for proper Myc expression and survival in the B cell, resulting in higher affinity B cell clones being selected [162]. The cycling of this process is referred to as affinity maturation and can result in significant genetic drift from the original BCR peptide sequence.

The switching of antibody classes is accomplished through the activity of AID within switch regions present throughout the constant region of the heavy chain locus. Downstream intronic promoters precede highly repetitive DNA sequences that make up the switch regions prior to each of the different heavy chain constant (C) domains (Fig. 3b). T cell dependent cytokine signaling initiates transcription of these promotors, giving AID access to the DNA sequence. Repair of AID deamination results in double-stranded breaks and allows the recombination between two switch regions, resulting in the complete removal of the intervening DNA by the process of non-homologous end joining. This brings the BCR V region in proximity to a new C gene, altering the antibody isotype. Note that the excised DNA is lost following repair and thus class switching is a unidirectional process.

### Class-switched antibodies

#### Immunoglobulin G (IgG)

IgG is the most abundant class of antibodies found throughout the body and is over half the antibody found in serum. IgG is divided into 4 subclasses in both humans (IgG1, IgG2, IgG3, and IgG4) and mice (IgG1, IgG2_a/c_, IgG2_b_, IgG3). IgG antibodies play a large variety of roles through the body, which includes complement activation, neutralization, opsonization of bacteria and cellular debris, and the induction of several immune cell functions through binding to various Fcγ Receptors. The ability of IgG molecules to complete each of these roles is dependent on the specific subclass of the antibody. The overall involvement of IgG antibodies in atherosclerosis is largely debated; however, the current consensus leans towards these molecules being atherogenic. Previously work has shown that treatment of *ApoE*^*−/−*^ mice with IgG from an atherosclerotic animal promotes atherosclerosis development [112, 163] and prevention of proper germinal center formation or function, either by blocking AID or CD40 signaling, has an atheroprotective effect [112, 160, 164]. Likewise, it has been shown that deletion of Fcγ receptors reduces atherosclerosis by preventing IgG-induced activation of vascular endothelial cells and the production of pro-inflammatory cytokines [165]. In humans, a meta-analysis of clinical studies suggested that patients with high levels of IgG are at increased risk for myocardial infarctions [127], while another analysis found the opposite [126]. Taken together, this data suggests that IgG function is harmful to plaque control and clearance.

While IgG may be atherogenic, IgG subclasses are not all the same, nor the IgG responses themselves. To get a better understanding of IgGs involvement in atherosclerosis, a more detailed look at the IgG subclasses should be performed. IgG1 is produced in response to IL-4 cytokine signaling and is the major IgG subclass observed following immunization and in *ApoE*^*−/−*^ mice [160]. Numerous immunizations have been performed in *ApoE*^*−/−*^ and *LDLR*^*−/−*^ mice over the years, and the effect of IgG1 on plaques has shown mixed results depending on the antigen. IgG1 responses to β2GP1, HSP60, GRP78, and cardiolipin showed increased plaque development in mice, while IgG1 against MDA-OxLDL, PC, and pCETP showed atheroprotection across mice, humans, and rabbits [135, 150, 166–171]. By comparison, mouse IgG2a/c in response to IFNγ signaling has been implicated as a major mechanism for the aggravation of atherosclerosis following IRA B-1 cell activation which activates macrophage through high-affinity binding to CD64 (FcγRI) [98, 172]. Meanwhile, IgG2b produced in response to TGFβ produced primarily by T_regs_, seems to have a protective role through binding of CD32b (FcγRIIb). Unlike the other Fcγ receptors which contain c-terminal cell-activating ITAM motifs, CD32b utilizes an inhibitory ITIM motif and has been shown to attenuate mice plaque formation, likely through the repression of inflammation and M2 macrophage polarization [173, 174]. This is further supported by a recently published paper by Ramiro and colleagues that found that IgG2b antibodies mounted against the self-antigen ALDH4A1 provides protection from plaque in *LDLR*^*−/−*^ mice on a high-fat diet [175].

#### Immunoglobulin E (IgE)

Of all the antibodies produced by B cells, IgE is the most unique in how it functions. Unlike IgM and IgG, IgE antibodies are found only at extremely low concentration within the serum and are instead found bound to the Fc epsilon receptor FcεRI expressed primarily on basophils, eosinophils, and mast cells. The binding of IgE to FcεRI is an extremely high-affinity interaction, occurring even in the absence of antigen, and is required for the proper effector function of these cells. Antigen binding to FcεRI-bound IgE induces cellular activation and the mass exocytotic release of many effector molecules, including IL-6, IL-13, granzyme B, histamine, antimicrobial peptides (LL37), and digestive peptidases (trypsin, cathepsin G, etc.) [176].

Mast cells promote inflammation, and while these cells can provide some regulatory function through the production of IL-10 [177], they are correlated with increased adverse clinical effects in human atherosclerosis and worsened plaque formations in mice [178, 179]. Thus, it is of little surprise that a plethora of clinical analyses have found IgE to be positively correlated with increased plaque burden and adverse events in both diabetic and nondiabetic individuals [179–184]. Furthermore, while only a few mice studies on IgE in atherosclerosis have been conducted so far, they tend to support this observation. In *ApoE*^*−/−*^ *IgE*^*−/−*^ mice, IgE was suggested to promote disease through the conversion of M2 macrophages into the M1 phenotype, encouraging foam cell development [185]. IgE was also found to be elevated in *LDLR*^*−/−*^*sIgM*^*−/−*^ mice, likely due to dysregulation of the CD23 negative feedback loop on B cells, which could have contributed to the increased plaque burden that was observed [186].

It is unknown what specifically triggers IgE production in atherosclerosis, nor do we know what most of the antigens are these antibodies target within the plaque. The most probable targets are reactive oxygen species-modified self-antigens released by necrotic cells. One study found IgE against the oligosaccharide allergen galactose-α-1,3-galactose (α-Gal) to have an increased correlation with coronary artery disease than total IgE levels in human cohorts. While the mechanism of these antibodies are uncertain, the authors suspect these antibodies may have emerged in response to α-Gal-glycosylated products alongside IgG1 [187]. Like IgG1, IgE is produced in response to IL-4; however, class switching to IgE, but not IgG1, can be inhibited by TGFβ, which may explain why these two antibody classes can show divergent involvement in plaque formation in some studies and similar effects in others depending on the target antigen.

#### Immunoglobulin A (IgA)

Research on IgA in atherosclerosis is difficult to complete in mice due to concern regarding our ability to translate any findings to humans because of differences in the biology of the two systems [188]. Humans have two IgA constant regions in the heavy chain locus compared to only one in mice. IgA likely also has additional biological functions in humans and other mammals that are not observed in mice due to mice lacking expression of FcαRI (CD89). In humans, IgA is found in high abundance in the serum, just after IgG, and is the most abundant antibody found in the mucosa. IgA is primarily a dimer linked by the Ig J chain and is the major antibody transported through endothelial cell by the FcRn protein. IgA is produced in response to the cytokines IL-5, IL-10, and TGFβ, and plays a critical role in the neutralization and clearance of a large range of environmental antigens.

In terms of atherosclerosis risk, very little is known about IgA. Several studies have discerned a correlation between serum IgA levels and atherosclerosis in terms of patient complications; although it is worth noting that the antigen-targets observed (PC and β2GP1) are strongly correlated with the disease in other Ig classes [171, 189–192]. IgA may provide protection in an epitope-specific manner as elevated anti-PC IgA seen in individuals from hunter societies in Kitava, as well as hibernating mammals, correlates with decreased incidence of cardiovascular disease, and suggests that immunological memory formed in response to infections may mediate the atherosclerotic risk associated with aging [151, 171, 193–195]. We recently reported that *ApoE*^*−/−*^ mice have slightly elevated serum IgA compared to C57BL/6 J animals, but this was minor compared to the differences we observed for IgM and IgG1 [160]. Regardless, no detailed study regarding the involvement of IgA in atherosclerosis has been done in mice to our knowledge.

## Conclusions

### Antibody dynamics: the importance of Isotype

Much of our understanding of antibodies in human atherosclerosis comes from meta-analysis of patient data which are almost entirely correlative in nature [126, 127, 171, 179–184, 189–192]. To investigate antibodies in a more mechanistic manner, hyperlipidemic mice deficient in the APOE or LDLR proteins have been routinely used. The most dramatic results have been seen following the breeding of these mice to mice where entire arms of B cell biology are ablated [106, 112, 160, 164, 196, 197]. This reductionist approach showed that B cells are important for modulating the disease as total loss worsened atherosclerosis while restricting the B cell compartment to B-1 cells aided disease attenuation. In addition, anti-OSE IgM antibodies are suggested to be able to control disease progression [61, 88–90, 108, 111, 160]. Interestingly, this neutralization and blocking of recognition by monocytes is maintained even when only the Fab domain of the antibodies were expressed [142, 157].

In *ApoE*^*−/−*^ mice, we observed a dramatic 6-fold increase in IgG1 levels, suggesting considerable B cell activation in these mice [160]. A recent study showed a 2.4-fold increase in plaque development by injecting 10 μg of *ApoE*^*−/−*^ IgG into Blimp1-deficient mice, which is required for proper plasma cell formation [112]. This suggest that the elevated IgG levels are atherogenic and may be involved in the propagation of the disease. Analysis of antigen specificity suggests that the *ApoE*^*−/−*^ IgG binds to self-proteins, with no substantial binding to oxLDL [160]. It was also observed that the antigen specificity has significant overlap between various isotypes, with 72% of antibody targets being shared between the IgM and IgG isotypes in both *ApoE*^*−/−*^ [160]. By comparison, C57BL6/J animals, which lack significant atherosclerosis compared to *ApoE*^*−/−*^ animals, share only 17% of IgM and 16% of IgG1 targets. Taken together, this may indicate that the generation of atherogenic autoreactive antibodies is dependent on specificity and less so isotype. While technically challenging, it would be interesting to see if the transfer of late-disease *ApoE*^*−/−*^ IgM into serum could also propagate disease. Likewise, we previously showed that *ApoE*^*−/−*^ *Aid*^*−/−*^ animals have high levels of MDA-oxLDL specific IgM [160]. To our knowledge, it has not yet been shown in the literature whether injection of late-stage *ApoE*^*−/−*^ antibody can overcome high titers of this protective antibody, or if these antibodies would instead block the detrimental effects as was observed with the *ApoE*^*−/−*^ IgG.

**Fig. 4:**
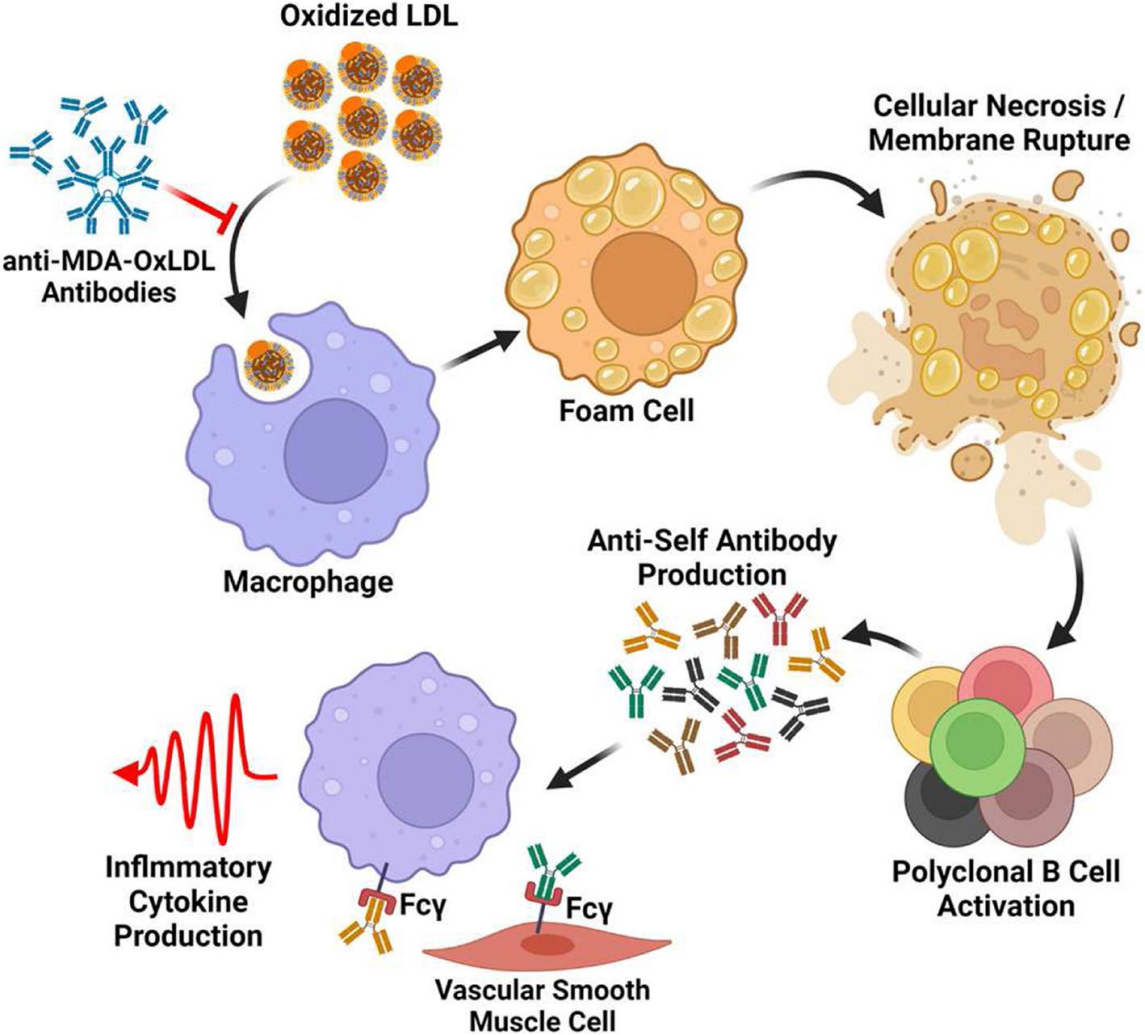
Proposed model for atherosclerosis development. Reactive oxygen species within arteries causes the oxidation of low-density lipoprotein (LDL). These molecules are bound by neutralizing MDA-OxLDL IgM antibodies, preventing the uptake of these molecules by macrophages. Imbalance between these antibodies and OxLDL results in lipid buildup within macrophages and the conversion to foam cells. These foam cells accumulate inside the intimal wall of the artery where they and cells of the surrounding tissue undergo necroptosis. This results in the release of intercellular antigens which may be modified by the plaque environment. These antigens trigger a polyclonal activation of B cells which form tertiary lymphoid organs and produce autoreactive antibodies. These antibodies then bind Fc receptors on macrophages, smooth muscle cells, and other cells of the innate immune system to produce inflammatory cytokines, propagating inflammation and disease progression

Based on these results and the current state of the literature, we propose the following model (Fig. 4). Anti-MDA-OxLDL and other OSEs antibodies produced by B-1 cells maintain normal tissue homeostasis. Decreases in this system due to aging or increases in LDL due to hyperlipidemia overwhelm this system, resulting in early plaque formation and the continuous uptake of OxLDL by macrophages. As the macrophage convert to foam cells, they accumulate in the intima, the resulting hypoxic environment promotes cellular rupture by necrosis and release of their cytosolic content. Intercellular proteins are modified by the plaque environment, permitting them to activate B cells which secrete self-reactive antibodies into the serum. These antibodies then bind Fc receptors expressed by myeloid and vascular smooth muscle cells, triggering the production of inflammatory cytokines and the propagation of disease.

#### Therapeutic potential of antibodies in atherosclerosis

The reason that the protection provided by OSE-specific antibodies is insufficient in hyperlipidemic mice and humans is not understood, but since loss of *Aid*^*−/*−^ seems to remediate this issue, we can speculate two possibilities [112, 160]. Inflammation-promoting class-switched B cells might outcompete protective B-1 cell antibodies due to consecutive signals present within the environment of advanced lesions and overwhelm the protective compacity of these cells. Another possibility is that AID activity in OSE-antibody-producing B cells results in genetic drift away from antibodies that can neutralize OSEs in favor of other epitopes present within the plaque environment.

We speculate that injecting mice, and by extension human patients, with MDA-OxLDL IgM antibody may provide attenuation of plaque burden and prevent disease progression in high-risk individuals. Several studies provide further support for this approach, showing that intravenous injection of Ig into *ApoE*^*−/−*^ and *LDLR*^*−/−*^ mice reduced plaques [128, 148, 198, 199]. Fascinatingly, two of these studies involved only IgG [198, 199]. This in combination with debates previously discussed highlights a major drawback of the reductionist approach used to understand this disease. The current dogma proposes IgM as protective and IgG as harmful, yet findings such as the discovery that anti-ALDH4A1 IgG2b is protective [175] illustrates that this oversimplification, if taken too literally, could be a detriment to our understanding. We suspect that antigen-specificity may play a more important role than isotype and hypothesize that transfer of MDA-OxLDL-specific IgG antibodies should also provide protection. To better translate our knowledge of this disease to the clinic, a closer look at the specific nature of the antibodies themselves and when they emerge needs to be focused. For example, it would be interesting to know how IgG that emerges in early plaques may differ from IgG observed in advanced lesions, and if these molecules could also have clinical benefit. While much ground has been covered, there is still much to be learned about how adaptive immunity impacts atherosclerosis.

## Data Availability

No supporting data was generated for this manuscript.

## Abbreviations

HDL: High-Density Lipoprotein
LDL: Low-Density Lipoprotein
OxLDL: Oxidized Low-Density Lipoprotein
OSE: Oxidation-Specific Epitope
DAMP: Danger-Associated Molecular Patterns
TCR: T Cell Receptor
MHC: Major Histocompatibility Complex
CD: Cluster of Differentiation
APC: Antigen Presenting Cell
T_H_: T Helper Cell (subclasses - T_H_1, T_H_2_,_ T_H_17)
IFN: Interferon
IL: Interleukin
T_reg_: T Regulatory Cell
TGFβ: Transforming Growth Factor Beta
ATLO: Artery Tertiary Lymphoid Organs
PVAT: Perivascular Adipose Tissue
BCR: B Cell Receptor
Ig: Immunoglobulin (Heavy chain subclasses – IgM: Mu; IgD: Delta; IgG: Gamma; IgE: Epsilon; IgA: Alpha; Light chains – Igκ: Kappa; Igλ: Lambda; Joining Chain - IgJ)
AID: Activation-Induced Deaminase
IRA: Innate Response Activator B Cells
GM-CSF: Granulocyte-Macrophage Colony-Stimulating Factor
MZ: Marginal Zone B Cells
FO: Follicular Zone B Cells
APOE: Apolipoprotein E
LDLR: Low-Density Lipoprotein Receptor
μMT: Mice unable to make IgM (and thus lack B cells)
C1q: Component 1 Subcomponent Q
C1qRp: Component 1 Q Subcomponent Receptor 1 (punitive)
PAMP: Pathogen-Associated Molecular Patterns
NK: Natural Killer Cell
Fc: Antibody Constant Domain (Crystallizable Fragment)
Fc [x]R: Antibody Receptor for specific Ig constant domains [FcαRI, Fcα/μR, FcμR, FcεRI, Fcγ]
sIgM^−/−^: Mice unable to secrete IgM
MDA: malondialdehyde
MAA: malondialdehyde-acetaldehyde
4HNE: 4-hydroxynonenal
PC: Phosphatidylcholine
POVPC: 1-palmitoyl-2-(5-oxovaleroyl)-sn-glycero-3-phosphocholine
PGPC: 1-palmitoyl-2-glutaroyl-sn-glycero-3-phosphocholine
PEIPC: 1-palmitoyl-2-(5(6)-epoxy-9-oxo-11-hydroxy-7E,14Z-prostadienoyl)-sn-glycero-phosphocholine
CuOxLDL: Copper-Oxidized Low-Density Lipoprotein
TUNEL: Terminal deoxynucleotidyl transferase dUTP nick end labeling
MERTK: MER Receptor Tyrosine Kinase
Fab: Fragment of Antigen Binding
C[x]: Antibody Constant Genes (See Ig)
β2GP1: Beta 2 Glycoprotein 1
HSP60: Heat-Shock Protein 60
GRP78: Glucose Regulated Protein 78
pCETP: Cholesteryl Ester Transfer Protein
ITAM: Immunoreceptor Tyrosine-based Activation Motif
ITIM: Immunoreceptor Tyrosine-based Inhibitory Motif
FcRn: Neonatal Fc Receptor
ALDH4A1: Aldehyde Dehydrogenase 4 Family Member A1
